# The first metatarsophalangeal joint in gout and asymptomatic hyperuricaemia

**DOI:** 10.1186/1757-1146-8-S2-O37

**Published:** 2015-09-22

**Authors:** Sarah Stewart, Nicola Dalbeth, Alain C Vandal, Keith Rome

**Affiliations:** 1 Health and Rehabilitation Research Institute, Auckland University of Technology, Auckland, 1142, New Zealand; 2 The University of Auckland, Auckland, 1142, New Zealand; 3 Ko Awatea. Counties Manukau Health, Auckland 2025, New Zealand

**Keywords:** gout, asymptomatic hyperuricaemia, first metatarsophalangeal joint

## Background

Gout is a painful arthritis associated with high serum urate levels (hyperuricaemia) and subsequent crystal deposition in joints. Although not all people with Asymptomatic Hyperuricaemia (AH) develop gout, crystal deposition and sub-clinical joint damage have been observed in this population. Gout most commonly affects the first metatarsophalangeal joint (1MTP). Existing theories suggest this may be related to the structure and functional role of the joint during gait, yet such characteristics of the 1MTP have not been investigated in people with gout or with AH. The objective of this study was to identify 1MTP characteristics in these people by comparing them with normouricaemic controls.

## Methods

Eighty-eight males (gout, n = 25; AH, n = 29; controls, n = 34) participated in this cross-sectional study. Primary outcome measures were 1MTP pain and passive 1MTP dorsiflexion. Secondary outcome measures were foot-related pain and disability and gait velocity. Exploratory outcome measures included additional patient-reported outcomes (general pain, patient global assessment, activity limitation and lower limb function), 1MTP plantarflexion and dorsiflexion muscle strength, hallux valgus severity, Foot Posture Index, 1MTP temperature, vibration perception threshold, plantar protective sensation and dynamic plantar pressure distribution (peak pressure and pressure time integral).

## Results

Compared to controls, people with gout had significantly reduced 1MTP dorsiflexion (p<0.0001), greater foot-related pain and disability (p<0.0001), reduced gait velocity (p=0.024), greater activity limitation (p=0.008), reduced lower limb function during activities of daily living (p=0.010) and recreational activities (p<0.0001), higher 1MTP plantar and dorsal temperature (p=0.033 and 0.034, respectively) and greater midfoot peak pressure and pressure time integrals (p<0.0001). Compared to controls, people with AH had significantly reduced lower limb function during recreational activities (p=0.033), higher Foot Posture Index (p=0.035), greater midfoot peak pressure and pressure time integral (p=0.001 and 0.002, respectively), greater first metatarsal peak pressure (p=0.029) and greater heel pressure time integrals (p=0.042).

## Conclusions

Gout negatively impacts foot-related patient-reported outcomes and is associated with impaired 1MTP function. The presence of hyperuricaemia in the absence of symptomatic gout is also associated with reduced lower limb function and may influence foot function during gait.

